# Pharmacological treatment of ambulatory schizophrenic patients in Belgium

**DOI:** 10.1186/1745-0179-2-11

**Published:** 2006-05-30

**Authors:** L Hanssens, M De Hert, M Wampers, J-Y Reginster, J Peuskens

**Affiliations:** 1 Service de santé publique, d'épidemiologie et d'économie de la santé, Université de Liège, Sart Tilman, 4000 Liège, Belgium; 2 University Psychiatric Center Katholieke Universiteit Leuven, Leuvensesteenweg 517, 3070 Kortenberg, Belgium

## Abstract

**Background:**

the objective of this study was twofold:

1) Describe the use of antipsychotic treatments in ambulatory patients suffering from schizophrenia in Belgium.

2) Evaluate to which extend antipsychotic treatment prescribing patterns are in accordance with published treatment guidelines.

**Method:**

A cross-sectional survey was carried out in 16 Belgian hospitals selected from a sample of 67 hospitals. The hospitals were equally distributed between the north and south part of the country and were representative of Belgian practice. During 2 months, participating psychiatrists were asked to record the medication use as well as demographic parameters of all consecutive ambulatory patients seen at their consultation or attending a day-hospital. Data concerning 1000 ambulatory patients with schizophrenia or schizoaffective disorder were collected.

**Results:**

In Belgium, the use of atypical antipsychotics is frequent (69%) in ambulatory patients with schizophrenia. In the overall sample, 73% receive only one antipsychotic drug. The majority of patients are treated with drugs of only one antipsychotic drug group, either first- typical (29.8%) or second-generation, atypical antipsychotics (53.2%). 15.8% of patients combine different types of antipsychotics. Antipsychotic dosing is adequate for the majority of patients but about one fifth receives a higher than recommended dose as per package inserts. Polypharmacy remains within reasonable limits. The use of concomitant medication varies according the antipsychotic treatment: patients who take second-generation antipsychotics only, receive the least additional drugs.

**Conclusion:**

Atypical antipsychotics appear to be the first line treatment for schizophrenic psychosis. Psychiatrists working with ambulatory patients are well aware of treatment guidelines and follow them quite adequately.

## 1. Background

Schizophrenia is a bio-psychosocial disorder where the interaction between an innate vulnerability and biological and psycho-social stressors determines whether an acute psychotic episode occurs or not [1-9]. The multidimensional nature of the disorder implies that treatment should focus on multiple aspects.

Although psychosocial and psychotherapeutic interventions are essential in optimising outcome, the biological treatment with antipsychotics is the cornerstone in the treatment of schizophrenic psychosis. Compliance with antipsychotic treatment is often a problem despite the proven effectiveness of these drugs. The burdensome side effects that regularly occur during treatment are an important reason for non-compliance beside factors inherent to the disease and fears of patients concerning the impact of long-term antipsychotic use [1,2].

To minimise the occurrence of these side effects, several guidelines have been formulated to optimise the use of antipsychotic medication in schizophrenic patients.

Second generation antipsychotics (atypical or "novel" antipsychotics, AAP) are regarded as the first line treatment for schizophrenic patients because of their broader symptomatological effectiveness in combination with a more favourable side effect profile (especially a decreased incidence of EPS) compared to first generation antipsychotics ("classical", typical antipsychotics, CAP) [3-8].

For both CAP and AAP, the occurrence of side effects, especially extra-pyramidal side effects, is related to the dose administered: the risk of side effects increases parallel to dose augmentations [9,10]. Adequate dosing is therefore recommended, especially since there is no evidence that higher doses will increase therapeutic effectiveness.

Since both CAP and AAP can give rise to specific side effects, combining them increases the risk of side effects and is hence advised against [3]. When different antipsychotics are combined medication schemes become more complex. This increases the risk of non-compliance since schizophrenic patients frequently suffer from severe cognitive deficits [11]. For the same reason, the use of concomitant medication should be kept to a strict minimum.

A number of studies focusing on antipsychotic prescription patterns have been conducted in several countries [9,12-14]. In analogy with these studies, our study aims at evaluating the use and dose of AAP in ambulatory patients with schizophrenia in Belgium. Additionally, we want to evaluate to which extend the medication use in ambulatory patients in Belgium is in accordance with the above mentioned treatment recommendations. Also, we wanted to compare the average daily dose used in ambulatory patients as opposed to the patients attending a day-hospital patients and describe co-medication usage

## 2. Methods

A cross-sectional survey was carried out in 16 Belgian hospitals, both university (n = 2) and non-university (n = 14) selected from a sample of 67 hospitals. The hospitals were equally distributed between the north and south part of the country. During 2 months (July and august 2003), participating psychiatrists were asked to record the medication use as well as demographic parameters of all consecutive ambulatory patients seen at their consultation or day-hospital.

The following parameters were collected:

Age, sex, last hospitalisation, years since ambulatory status, diagnosis, co-morbidities, GAF score, CGI, age at first hospitalisation, level of education, living situation, occupational status, psychiatric and somatic medication, laboratory assessments, ECG, cognitive evaluation and numbers of visits to the general practitioner.

The study was approved by the ethical committee of the University Centre Sint-Jozef, Kortenberg, Belgium.

All patients with a DSM 295.xx diagnosis were included. Each clinician included 21 patients on average

The following aspects of medication use were evaluated:

Firstly, it was evaluated if and to which extend CAP and AAP are being prescribed in combination (Combination therapy). Two forms of combination-therapy can be distinguished: on the one hand a combination of AAP and high potency CAP, on the other hand a combination of AAP and low potency CAP.

Secondly, we evaluated the prevalence of monotherapy. Two meanings of the concept "monotherapy" can be distinguished: monotherapy can refer to the use of drugs of only one antipsychotic class, either AAP or CAP, irrespective of the number of different drugs administered. In the remaining part of the text, we will refer to this form of monotherapy with the labels "Only AAP" and "Only CAP".

A second meaning of monotherapy refers to the use of only one antipsychotic agent, we will refer to this definition as "real monotherapy". For the total study sample and for each of the treatment conditions defined above, the occurrence of actual monotherapy and the number of different antipsychotics that are prescribed simultaneously, as well as the concomitant medication administered, was evaluated. The average daily dose administered for CAP and AAP separately was also assessed.

Descriptive statistics were applied for basic demographic and clinical data as well as for data on medication usage. Comparisons between antipsychotic treatment groups or between patients groups for continuous variables are calculated by means of an ANOVA. For categorical data a chi-square test was used.

## 3. Results

### 3.0 Participating sites

The overall geographical distribution of the sites does not differ from the National distribution from the National Institute of Health. Data from 1000 patients were collected by the 16 sites

### 3.1. Patient characteristics

From the 1000 patients with a DSM-IV 295.xx diagnosis, 737 are treated ambulatory and 263 are attending a day hospital but live in the community.

Paranoid schizophrenia is the most frequently observed diagnosis (58.2%), followed by schizoaffective disorder (16.3%) and undifferentiated schizophrenia (12.5%). The other subtypes were present in less than 10% of patients. 7% of patients have an additional diagnosis on axis I, 16% on axis II. The majority of these patients are male (64.1%) and unemployed (79.5%). The mean age in the population is 40.5 years (std11.6). Patients have been ill for 14.7 years (std 10.4) on average and have on average been admitted 5 times (std 4.3).

Patients' mean GAF-score was 62 (std 12.3) [15]. 36.4% of patients have a CGI of moderately ill, another 21.8% have more severely ill CGI-scores [16]. The correlation between GAF and CGI is -0.87 (p < .0001).

At the time of assessment, half of the patients (50.9%) had been treated ambulatory for more than two years.

### 3.2. Medication use and distribution

#### 3.2.1. Use of antipsychotics

On average, patients receive 1.3 different antipsychotic drugs (std 0.6). The prevalence of the predefined treatment conditions is shown in table 1.

**Table 1:** Antipsychotic treatment condition in ambulatory schizophrenic patients.

	% patients
No AP or trial medication	1.2
Only CAP	29.8
Only AAP	53.2
Combination high potency CAP with AAP	9
Combination low potency CAP with AAP	6.8

From the above table, we can see there is a high usage of AAP in this population: 69% of patients are treated with AAP. One fourth (24.7%) of CAP are used for sedation (as indicated by the clinician, and in the analysis also based on type of CAP and dose).

37.9% of CAP are administered through depot injections. About one quarter (24.2%) of the patients treated with depot injections use additional AAP. In the total population, 21.5% of the patients are treated with depot injections.

As can be seen in table 2, real monotherapy, i.e. the use of one and only one antipsychotic, is observed in almost three quarters of patients (73%). Only 4.2% of patients combine more than two different antipsychotics.

**Table 2:** Number of different AP.

% Patients	Number of AP (AAP + CAP)			
	
	1	2	3	4
Only AAP	93.8	6.2	0	0
Combination low potency CAP with AAP	0	86.8	13.2	0
Combination high potency CAP with AAP	0	78.9	16.7	4.4
Only CAP	74.5	20.8	4.4	0.3

Total population	73	22.8	3.7	0.5

**Figure 1:**
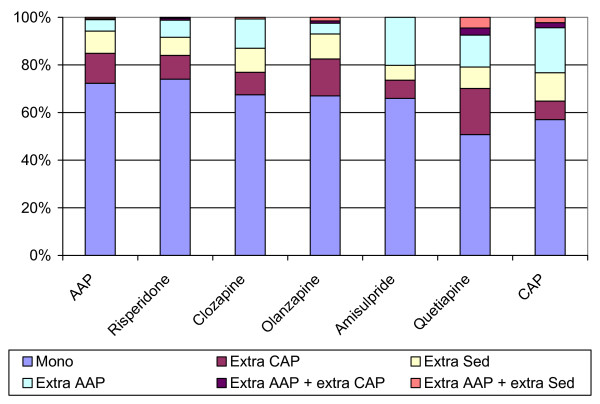
Combination of different AP.

The proportion, in which the different AAP are used in monotherapy, is comparable for most AAP and is about 70%. As opposed to the other drugs, quetiapine is used in monotherapy in about 50% of the patients (Figure 1).

Risperidone is the most frequently used AAP used by 34.4% of patients treated with AAP. Olanzapine is used by 27.4%, clozapine by 18.9%, quetiapine by 9.2%, amisulpride by 8.9%, sertindole by 0.7 and aripiprazole by 0.6% of patients prescribed AAP (both aripiprazole and sertindole were not commercially available at the time of the study).

#### 3.2.2. Antipsychotic doses

##### 3.2.2.1. Dose of Typical Antipsychotic (AP)

Table 3 gives the average daily CAP-dose per patient expressed in haloperidol equivalents. To evaluate the occurrence of overdosing, we calculated the percentage of patients being treated with doses below, within or above the advocated dose range of 5 to 10 mg haloperidol equivalents^8^. 49.5% of patients receive a low dose of CAP (mean 2.3 mg, std 1.3) and 19.5% of patients get a higher than recommended dose of CAP (mean 18.8 mg, std 9.6).

**Table 3:** Mean equivalent dose per patient for CAP.

	Mean equivalent Dose	STD	N
High potency CAP	6.8	6.3	371
Low potency CAP	1.1	0.6	131
All CAP	6.0	6.2	456

##### 3.2.2.2. Dose of Atypical Antipsychotic use

**Figure 2:**
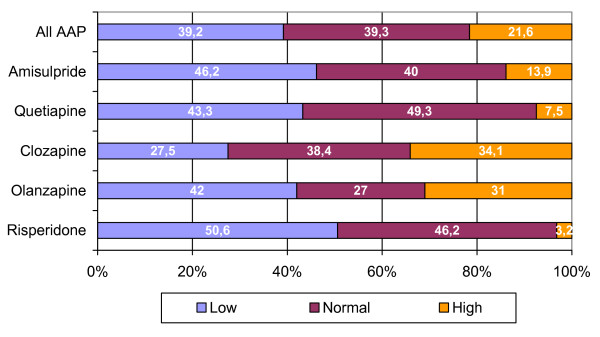
Frequency (%) of high, low and normal doses for the most frequently used AAP.

For the most frequently used AAP and for the total group of AAP, average daily doses for each of the defined dose ranges are given in table 4. The occurrence of under- and overdosing, relative to the label instructions, is displayed in figure 2.

**Table 4:** Mean daily dose per patient for the most frequently used AAP.

	Mean dose	STD	N
Olanzapine	14.9	6.9	200
Risperidone	3.7	1.8	251
Clozapine	286.4	146.5	138
Quetiapine	600	249.2	67
Amisulpride	596.2	269	65

#### 3.2.3. Concomitant medication use

The patients of this sample use 2.97 (std 1.8) different drugs on average of which 1.7 (std 1.6) are psychotropic agents and somatic medication.

One third of the patients (33%) are treated with more than three different drugs. Psychotropic medications other than antipsychotics are used by 68.7% of patients and 28% receive somatic medication. The frequency with which different categories of psychotropic medication are used is shown in table 5.

**Table 5:** Use of concomitant psychotropic medication.

Psychotropic medication	% patients using a drug from a medication group
Anticholinergics	21.6
Benzodiazepines	36.2
Mood stabilisers	15.2
Antidepressants	39.1

**Figure 3:**
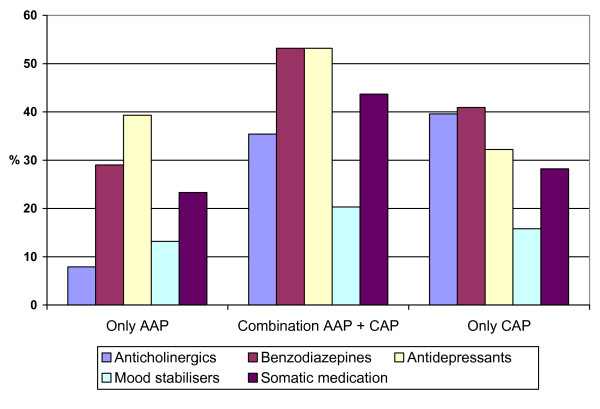
Use of concomitant medication as a function of antipsychotic treatment.

The total number of additional psychotropic medication patients use is significantly influenced (p < .001) by antipsychotic treatment. The use of benzodiazepines (p < .01), anticholinergics (p < .01) and antidepressants (p < .01) are all influenced by the type of antipsychotic treatment as can be seen in Figure 3.

Sedative CAP are combined with benzodiazepines in 8% of patients.

**Figure 4:**
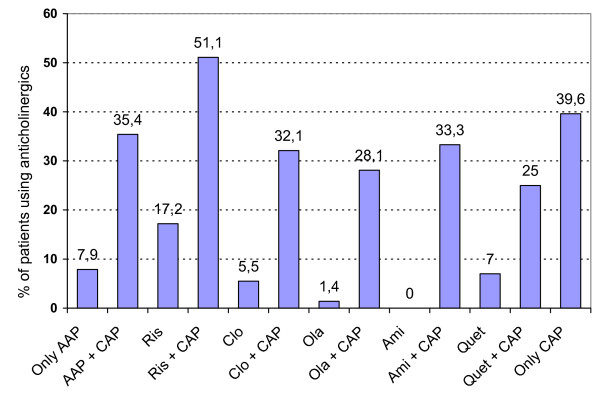
Use of anticholinergics (%) as a function of antipsychotic treatment.

The use of anticholinergics as a function of antipsychotic treatment is specified in table 6 and figure 4.

**Table 6:** Use of anticholinergics as a function of antipsychotic treatment.

Antipsychotic treatment	% of patients using anticholinergics
Only AAP	7.9_a_
Combination sedative CAP with AAP	26.5_b_
Combination incisive CAP with AAP	42.2_b_
Only CAP	39.6_b_

a, b: treatment conditions with a different subscript differ at .05-level

From figure 4 and table 6 it appears that the advantage of AAP regarding EPS vanishes when AAP are combined with CAP. Even when AAP are combined with sedative CAP, anticholinergic use increases. The negative influences of adding CAP to AAP is also observed when the most frequently used AAP are considered separately (Figure 4).

Patients with schizophrenia additionally receive 0.3 (std 0.5) somatic drugs on average. Almost three in four patients (72%) do not take any somatic drugs. Women take significantly more somatic drugs than men (p < .0005). Only 7.8% of female patients use contraceptive medication. Somatic drug use increases with age (p < .0005). 3% of patients are treated for diabetes, 1.3% for lipid abnormalities.

### 3.3. Clinical care

Every 5.4 weeks on average, ambulatory patients are in contact with their psychiatrist. Patients in day care are in contact with the treatment team 3.4 days a week on average. Ambulatory patients less frequently (Mean 0.7 visits) contact a medical specialist than patients in day care (Mean 1.2 visits) (p < .05).

Almost half of the patients (48.6%) do not have a GP. This problem is more pronounced in day care patients (61.6%) as compared to ambulatory patients (44%). Annually, ambulatory patients see their GP more frequently (Mean 3.3 visits) than patients in day care (Mean 1.9 visits) (p < .005). Patients on depot medication more frequently see their GP than patients not treated with depot medication (p < .0005).

Female patients more frequently visit their GP than male patients (p < .0005). The number of visits to a GP significantly (p < .0005) increases with age.

About one fourth of patients have an ECG performed in the past 6 months. Cognitive functioning is evaluated in 7.5% of patients and only 39.6% undergo a laboratory assessment in the last 6 months (including compulsory haematological assessment in patients treated with clozapine). Regular screening for metabolic side-effects is only performed in 3 sites.

### 3.4. Differences between ambulatory patients and patients in day care

#### 3.4.1. Demographic and clinical characteristics

With respect to diagnosis, gender and educational level, there are no significant differences between patients treated ambulatory and patients in day care. Compared to ambulatory patients, patients in day care more frequently are unemployed (p < .01).

Patients in day hospital (mean age 39.3 years, std 12.4) are significantly younger than ambulatory patients (mean age 41 years, std = 11.5). Ambulatory patients have a significantly (p < .0005) higher GAF-score (Mean 64.3) than patients in day hospital (Mean 55.8).

The CGI-scores are distributed significantly different (p < .0001) for ambulatory patients and patients in day hospitalisation, ambulatory patients are less severely ill.

The duration of ambulatory care since last hospitalisation is significantly longer than the duration of treatment in day care since last hospitalisation which might suggest a more stable condition in ambulatory patients.

#### 3.4.2. Antipsychotic medication

**Figure 5:**
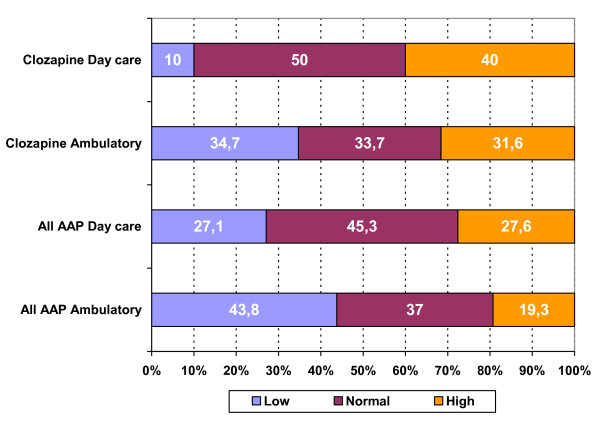
Frequency (%) of now, high, and normal dosing for clozapine and all AAP.

The distribution of patients over the different antipsychotic treatments defined is significantly different for ambulatory patients and patients in day care (p < .01). Compared to ambulatory patients, patients in day hospitalisation less frequently use olanzapine and clozapine in monotherapy (p < .05). For some of the AAP as well as for the group of AAP as a whole, dosing is significantly higher in the day care group (Table 7). For clozapine and the group of AAP in general the frequency of over- and under-dosing also differs as a function of patient group (p < .05) (Figure 5).

**Table 7:** Dosages AAP per patient group.

		Mean Dose	STD	N	p
Olanzapine	Ambulatory	14.3	6.5	146	p < .05
	Day Care	16.8	7.4	54	
	Total	14.9	6.9	200	
Risperidone	Ambulatory	3.7	1.9	188	ns
	Day Care	3.8	1.5	63	
	Total	3.7	1.8	251	
Clozapine	Ambulatory	269.4	154	98	p < .05
	Day Care	328.1	117.6	40	
	Total	286.4	146.5	138	
Quetiapine	Ambulatory	582.1	267.4	39	ns
	Day Care	625	223.8	28	
	Total	600	249.2	67	
Amisulpride	Ambulatory	572.8	266.6	46	ns
	Day Care	652.6	273.6	19	
	Total	596.2	269	65	

#### 3.4.3. Concomitant medication

Outpatients (Mean 2.8, std 1.7) take significantly less different drugs than patients in day care (Mean 3.4, std 2). Patients in day care more frequently (27%) use anticholinergics than ambulatory patients (19.7%) (p < .05). Somatic medication is also used significantly less frequently by ambulatory patients. Female patients in day care more frequently use oral contraception.

## 4. Discussion

In the total sample, representing ambulatory and day-care patients, almost three in four patients (73%) are treated with one antipsychotic: this percentage is higher in the groups of patients only treated with AAP (93.8%) as compared to patients only receiving CAP (74.5%). Obviously, patients on combination therapy receive at least two different antipsychotics.

In ambulatory patients in Belgium, AAP are frequently used: 69% of patients are treated with AAP either in monotherapy, or in combination with CAP. Second generation antipsychotics represent the majority prescribed antipsychotics. The percentage of patients treated with CAP is clearly lower: 45.6%.The most common antipsychotic treatment is monotherapy of AAP: 53.2%. Monotherapy of CAP occurs in 29.8% of patients. Only 15.8% receive a combination of AAP and CAP. These results are in-line with a recent comparable study carried out in France which included 1861 ambulatory patients [17].

For both AAP and CAP, average daily doses lie within the advocated dose range. The majority of the patients receive drug doses that fall under or within the advocated dose range. Dosing is similar in both patients groups, when differences are observed, day care patients receive the highest doses. Higher than recommended doses are being used in about 20% of patients, both for CAP and AAP.

Beside antipsychotic medication, patients on average receive 1.7 additional drugs. Day care patients receive more concomitant medication than outpatients. Especially the use of anticholinergics and somatic drugs appears increased in the former group. The low prevalence of adequate contraception in female patients is problematic.

In the total population, the use of additional medication appears to be influenced by antipsychotic treatment. Patients only treated with AAP generally need less concomitant medication than patients in other treatment conditions. Anticholinergics in particular are used significantly less by patients only treated with AAP. If AAP are combined with CAP, even only for sedation, the use of anticholinergics increases significantly. This clearly indicates that the EPS advantage of AAP disappears when AAP are combined with CAP. This confirms that AAP are the first line treatment choice for schizophrenic psychosis.

The percentage of patients treated for diabetes and lipid abnormalities is fairly low suggesting under-diagnosing corroborated by the fact that systematic screening for metabolic disorders is restricted to a limited number of sites. This is in agreement with recent surveys still indicating low awareness of clinicians about metabolic side-effects of antipsychotic medication, while there is a large body of evidence suggesting that people with schizophrenia are a high-risk group for the development of metabolic abnormalities [18-24].

Overall the somatic follow-up of patients with schizophrenia could be improved by active involvement of GP's. About 50% of patients studied do not have a GP.

Cognitive evaluation and ECG-monitoring are not done on a routine basis in clinical care.

In general, there are no important differences between outpatients and patients in day care. As can be expected, day care patients are more severely ill than outpatients. Other differences between both patient groups, like the increased use of concomitant medication and higher dose of antipsychotic medication by day hospitalisation patients, can be interpreted as a consequence of this difference in illness severity.

Although progress has been made in the therapeutic care of patients with schizophrenia and treatment is being provided on an ambulatory basis for the majority of patients in Belgium, most of them remain unemployed and dependent on social disability incomes which constitutes a high societal burden [25]. Efforts should be made to both support adequate treatment choices as well as overall care including social integration

## 5. Conclusion

Our results show that Belgian psychiatrists treating ambulatory patients are well aware of guideline recommendations on the use of antipsychotic medication, and generally follow them quite adequately. The use of AAP with their broader symptomatological efficacy and more favourable side effect profile appears well established in clinical practice: 69% of patients are treated with AAP. The large majority of patients (73%) are treated with only one antipsychotic. Actual monotherapy is even more frequent (93.8%) when patients are only treated with AAP.

The latter group also receives less concomitant medication than patients in other treatment conditions. Especially anticholinergic use is less frequent in patients only treated with AAP. This finding indirectly suggests less EPS in patients only treated with AAP.

Medication schemes are hence simplest in this patient group which is expected to positively influence compliance. Dosing for both CAP and AAP generally falls within the advocated dose ranges.

At the time of the study there was limited awareness in clinicians about metabolic side-effects of antipsychotic medication.

## Competing interests

The author(s) declare that they have no competing interest.

## Authors' contributions

Study planning and design: M. De Hert, L. Hanssens, J. Peuskens

Data collection and statistical analysis: M. De Hert, J-Y. Reginster, M. Wampers

Drafting report: all

## References

[B1] Baldessarini RJ Cohen BM Teicher MH Significance of neuroleptic dose and plasma level in the pharmacological treatment of psychoses Arch Gen Psychiatry 1988 45 79 91 2892478 10.1001/archpsyc.1988.01800250095013

[B2] Kulkarni J Power P McGorry P, Jackson H Initial treatment of first-episode psychosis The recognition and management of early psychosis 1999 Cambridge: University Press 184 205

[B3] NICE (National Institute for Clinical Excellence) Guidance on the use of newer (atypical) antipsychotic drugs for the treatment of schizophrenia 2002 London: NICE

[B4] Davis JM Chen N Glick ID A meta-analysis of the efficacy of second-generation antipsychotics Arch Gen Psychiatry 2003 60 553 564 10.1001/archpsyc.60.6.553 12796218

[B5] De Hert M Peuskens J Schizofrene psychose Welzijnsgids – Gezondheidszorg, Geestelijke Gezondheid 2002 161 186

[B6] Kane JM Leucht S Carpenter D Docherty JP Expert consensus guideline series. Optimising pharmacologic treatment of psychotic disorders. Introduction: methods, commentary and summary J Clin Psychiatry 2003 Suppl 12 5 19 14640142

[B7] Lieberman JA Koreen AR Chakos M Sheitman B Woerner M Alvir JM Bilder R Factors influencing treatment response and outcome of first-episode schizophrenia: implications for understanding the pathophysiology of schizophrenia J Clin Psychiatry 1996 Suppl 9 5 9 8823344

[B8] Peuskens J De Hert M Good medical practice antipsychotics 1999 Copenhague: Lundbeck

[B9] Emsley RA Oosthuizen PP Joubert AF Roberts MC Stein DJ Depressive and anxiety symptoms in patients with schizophrenia and schizophreniform disorder J Clin Psychiatry 1999 60 47 751 10.4088/jcp.v60n1105 10584762

[B10] McEvoy JP Hogarty GE Steingard S Optimal dose of neuroleptic in acute schizophrenia. A controlled study of neuroleptic threshold en higher haloperidol dose Arch Gen Psychiatry 1991 48 739 745 1883257 10.1001/archpsyc.1991.01810320063009

[B11] Robben N De Hert M Peuskens J Gebrek aan ziekte-inzicht bij schizofrene patiënten Tijdschrift voor Psychiatrie 2002 44 313 322

[B12] Harrington M Lelliott P Paton C Konsolaki M Sensky T Okocha C Variations between services in polypharmacy and combined high dose of antipsychotic drugs prescribed for in-patients Psychiatric Bulletin 2002 26 418 420 10.1192/pb.26.11.418

[B13] Janssen F Peuskens J D'haenens M De Hert M Hulselmans J Meire I Geheugenstoornissen bij schizofrene patiënten Neuron 2001 Suppl 6 2 10

[B14] Lelliott P Paton C Harrington M Konsolaki M Sensky T Okocha C The influence of patient variables on polypharmacy and combined high dose of antipsychotic drugs prescribed for in-patients Psychiatric Bulletin 2002 26 411 414 10.1192/pb.26.11.411

[B15] Jones SH Thornicroft G Coffey M Dunn G A brief mental health outcome scale-reliability and validity of the Global Assessment of Functioning GAF Br J Psychiatry 1995 166 654 659 7620753 10.1192/bjp.166.5.654

[B16] Guy W E.C.D.E.U. Assessment manual for psychopharmacology 1976 Rockville MD: Department of Health, Education and Welfare

[B17] Blin P Olie JP Sechter D Petitjean F Cialdella P Gerard A Hanssens L Westerloppe J Utilisation des neuroleptiques chez les patients ambulatoires souffrant de schizophrénie Rev Epidemiol Santé Publique 2005 53 601 613 10.1016/s0398-7620(05)84740-6 16434933

[B18] De Hert M Van Eyck D Peuskens H Thys E Wampers M Scheen A Peuskens J Oral Glucose Tolerance Test in treated patients with schizophrenia: Data to support an adaptation of the proposed guidelines for monitoring patients on second generation antipsychotics Eur Psychiatry 2005 available online 16139484 10.1016/j.eurpsy.2005.05.011

[B19] Buckley PF Miller DD Singer B Clinicians' recognition of the metabolic adverse effects of antipsychotic medications Schizophr Res 2005 79 281 288 10.1016/j.schres.2005.04.010 15964743

[B20] Newcomer JW Nasrallah HA Loebel AD The atypical antipsychotic therapy and metabolic issues national survey J Clin Psychopharmacol 2004 24 Suppl 1 6 10.1097/01.jcp.0000142281.85207.d5 15356414

[B21] Newcomer JW Second-generation (atypical) antipsychotics and metabolic effects: a comprehensive literature review CNS Drugs 2005 19 Suppl 1 1 93 15998156 10.2165/00023210-200519001-00001

[B22] Scheen AJ De Hert M Drug-induced diabetes mellitus: the example of atypical antipsychotics Revue Med Liege 2005 60 5–6 455 60 16035311

[B23] American Diabetes Association Consensus Development Conference on Antipsychotic Drugs and Obesity and Diabetes Diabetes Care 2004 27 596 601 14747245 10.2337/diacare.27.2.596

[B24] De Hert M van Winkel R Van Eyck D Hanssens L Wampers M Scheen A Peuskens J Prevalence of the metabolic syndrome in patients with schizophrenia treated with antipsychotic medication Schizophr Res 2006 83 1 87 93 10.1016/j.schres.2005.12.855 16481149

[B25] De Hert M Thys E Boydens J Gilis P Kesteloot K Verhaegen L Peuskens J Health expenditure on schizophrenic patients in Belgium Schizophr Bull 1988 24 4 519 527 10.1093/oxfordjournals.schbul.a033346 9853786

